# “Studying cognitive reappraisal as an antidote to the effect of negative emotions on medical residents’ learning: a randomized experiment”

**DOI:** 10.1186/s12909-022-03996-2

**Published:** 2023-01-28

**Authors:** Telma Kremer, Silvia Mamede, Maria P. T. do Nunes, Walter W. van den Broek, Henk G. Schmidt

**Affiliations:** 1 Institute of Medical Education Research Rotterdam, Erasmus Medical Centre, Rotterdam, The Netherlands; 2 Department of Psychology, Education and Child Studies, Erasmus University Rotterdam, Rotterdam, The Netherlands; 3 Department of Internal Medicine, São Paulo University Medical School, São Paulo, Brazil

**Keywords:** Emotions, Learning, Medical education, Cognitive reappraisal

## Abstract

**Background:**

Medical trainees often encounter situations that trigger emotional reactions which may hinder learning. Evidence of this effect on medical trainees is scarce and whether it could be counteracted is unclear. This study investigated the effect of negative emotions on medical residents’ learning and whether cognitive reappraisal counteracts it.

**Methods:**

Ninety-nine medical residents participated in a three-phase experiment consisting of: (1) *watching a video,* either a neutral or an emotion-induction version, the latter either followed by cognitive reappraisal or not (2) *learning*: all participants studied the same medical text; study-time and cognitive engagement were measured; (3) *test*: a recall-test measured learning. Data was analysed using Chi-square test and one-way ANOVA.

**Results:**

Study time significantly varied between conditions (*p* = 0.002). The two emotional conditions spent similar time, both significantly less than the neutral condition. The difference in test scores failed to reach significance level (*p* = 0.053). While the emotional conditions performed similarly, their scores tended to be lower than those of the neutral condition.

**Conclusion:**

Negative emotions can adversely affect medical residents’ learning. The effect of emotions was not counteracted by cognitive reappraisal, which has been successfully employed to regulate emotions in other domains. Further research to examine emotion regulation strategies appropriate for medical education is much needed.

**Supplementary Information:**

The online version contains supplementary material available at 10.1186/s12909-022-03996-2.

## Background

Emotionally difficult situations are unavoidable in medical training. Students and doctors have reported experiencing negative emotions such as sadness when watching a patient’s suffering, fear of making mistakes, or anger when witnessing colleagues’ unprofessional behaviour [1, 2]. Transitioning from undergraduate education to residency training is a challenging moment, yet how residents deal with negative emotions has rarely been investigated [3]. Although psychological research has examined the influence of emotions on cognitive processes, studies with medical trainees are still rare [4–9]. Several authors have recently called for attention to the problem, considering the preliminary evidence that emotional responses can hinder residents’ [10] and medical students’ acquisition of new knowledge [6]. The residency training is a period of intensive learning, and if commonly experienced negative emotions hamper what residents learn during this time, opportunities for improving competence could be missed. It is therefore crucial to develop and test interventions that can help residents regulate their emotions when they arise. The present study aimed to contribute to this goal.

How could emotions affect residents’ learning? Research in cognitive psychology has described several mechanisms through which both positive and negative emotions can interfere with cognitive processes involved in learning. For example, positive emotions are known to trigger a “global processing” style that makes it easier to see the “big picture” in a situation and to increase learners’ ability to think flexibly [6]. Negative predispositions triggered by an emotionally difficult situation can be automatically activated by a similar one, leading the learner to attempt to avoid (rather than engage with) it [11]. Another possible way of disruption could happen when part of the learner’s cognitive resources is captured to process emotions, leaving less available ones to process new learning material [12, 13].

In the residency training, emotions can interfere with cognitive processes, such as attention and perception, for example, when a resident studies an online material about a patient’s problem. If attention is deviated to other tasks such as processing of emotions, integration of the to-be-learned information into knowledge structures stored in memory will suffer [10]. Emotional information relevant for the task at hand tends to be better retained in memory [14], but this happens at the expenses of the neutral information processed concomitantly, which becomes therefore less available for future retrieval [15]. There is some empirical evidence of the negative impact of emotions on residents’ and students’ learning. Kremer and colleagues [10] found residents under the influence of negative emotions triggered by a videoclip to subsequently spend less time when studying a medical text and to recall less of the text content. McConnell and colleagues [9] observed that even mild affective states can impair undergraduate students’ learning of basic science concepts. Moreover, negative emotional states have also been shown to decrease interest and motivation to engage with learning materials [7].

This evidence points to the importance of assisting residents in dealing with commonly experienced emotions, thereby allowing them to benefit from learning opportunities. Possibly relevant here are strategies that have been investigated by research on Emotion Regulation. A well-known model of Emotion Regulation (ER) [16] defines five overlapping categories of emotion regulation strategies, which aim at changing the emotion-triggering situation by avoiding it, by focusing on the task on hand (distraction), by hiding emotions (suppression), by actively modifying the situation or by reframing the situation into a more positive perspective. Previous research suggests that medical trainees commonly use distraction and suppression [17, 18]. Although effective in helping complete the tasks at hand, they seem maladaptive strategies, potentially entailing high long-term costs both for competence development and personal well-being [18–20]. Physicians usually face situations in which it is important to be aware of, and eventually learn from, emotionally charged aspects of interactions. Taken together, these findings highlight the importance of finding more suitable ER interventions.

One promising intervention seems to be *cognitive reappraisal* [16], a particularly well-studied form of *cognitive change* which involves “modifying one’s appraisal of a situation in order to alter its emotional impact” [21]. Although cognitive reappraisal can be used to increase or decrease both negative and positive emotions, it is most frequently employed to decrease negative emotional reactions. By engaging in cognitive reappraisal one can see a negative experience in a different light, thereby changing its meaning or personal relevance [20–22]. For instance, a student may reframe failing an exam as an opportunity to improve and do better in future tests or re-evaluate the impact of that failed exam as not entirely precluding his/her chance of succeeding in the course. Reappraising a difficult experience seems to reduce its potential adverse impact. For example, in an experiment on regulation of aversive emotions elicited by videoclips [23], participants who used cognitive reappraisal reported less distress and showed less physiological responses relative to those who simply watched the same videoclips. Cognitive reappraisal has additional advantages: it demands relatively little investment, is always available and may have better cost–benefit than other emotion regulation strategies [20, 22]. However, as far as we know, it has not been tested as a tool to help medical residents deal with negative emotions.

The present study had a two-folded purpose. We examined (i) whether negative emotions hinder learning of scientific information, and (ii) whether this impact (if any) was counteracted by an intervention based on cognitive reappraisal. Residents watched either a neutral or an emotional videoclip, the latter either followed by cognitive reappraisal or not. Subsequently, all residents studied a relevant medical text and took a recall test. Consistently with the study purpose, the main outcome measurement was test score. To get insight on the residents’ engagement with the learning task, we also measured cognitive engagement and study time. Based on previous research [10], we expected: (a) time invested in studying the text and cognitive engagement in this task under the emotional-with-antidote condition would both be similar to the neutral condition and higher than the emotional-without-antidote condition; (b) test scores under the emotional-with-antidote condition would be similar to the neutral group, both higher than the emotional-without-antidote condition.

## Methods

### Design

**Fig. 1:**
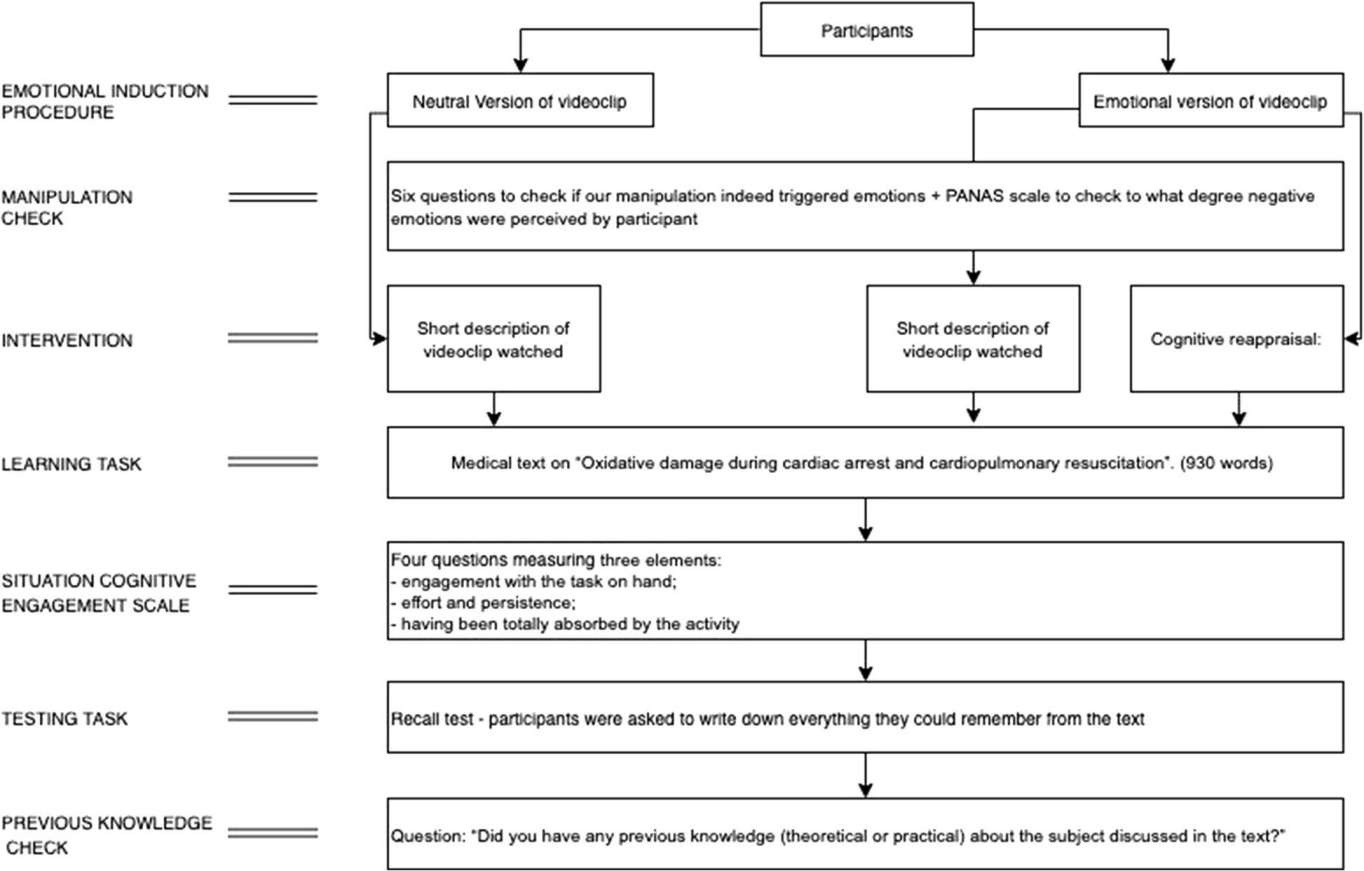
Experiment design and tasks flow

This study was an experiment consisting of three tasks: (I) an *emotion induction procedure*, in which participants watched a short videoclip under one of three conditions: neutral, emotional-without-antidote, emotional-with-antidote, (from now on called N, E and E-CR); (II) a *learning task*, in which all participants studied the same medical text, and study time and situational cognitive engagement (SCES) were measured; (III) a *test*, in which a recall test measured learning of the content of the text (Fig. 1).

### Participants

Participants were ninety-nine first-year internal medicine residents^1^ at São Paulo University Medical School, Brazil. The study was conducted during a ‘bootcamp’ in the 2020 welcome week of the residency programme. All 99 residents who participated in the welcome week accepted to voluntarily and anonymously participate in the study. As the nature of the study prevented prior disclosure of its objectives, participants were informed about their tasks before the study and debriefed later.

All participants signed informed consent forms. The study protocol was approved by the Research Ethics Committee of the São Paulo University (659.347 in 21/05/2014).

### Materials and procedure

The study was presented to the participants as two separate, unrelated studies, from two different institutions. The emotion induction procedure was presented as a study on the use of fictitious videoclips in medical education whereas the learning task and the test were purportedly a study on the suitability of new medical discoveries to residents’ education. This procedure was used to prevent participants knowing that we were studying the effect of emotions on learning, which could trigger attempts to control that effect [24].

Before the study started, participants were randomly assigned (www.random.com/NA MEDIA®/2016) to one of the three conditions (N, E, E-CR), defined by the videoclip used (either neutral or emotional) and the subsequent task (either reading a description of the video or following instructions for cognitive reappraisal) (Fig. 1). Participants of each condition were tested in separate auditoriums under the surveillance of one researcher or research assistant.

#### Emotion induction procedure

We opted to use a vicarious experience to induce negative emotions for ethical reasons. Experimental research has commonly employed films to elicit standardized emotional responses [6, 24]. We used a 3-min videoclip, produced to closely resemble a real situation (e.g. the actors were physicians dressed as usually and the medical files handled used the hospital stationary paper), showing a committee of senior clinicians interviewing a resident about a critical incident at the ER where a patient died. In the emotional version (E and E-CR), the committee used an accusatory and harsh approach, and the resident in the videoclip showed clear signs of being upset, afraid and anxious. Conversely, in the neutral version (N), the approach was respectful and supportive, and the committee showed to consider the difficult circumstances in the ER at the time. (Fig. 1). The videoclips were used in a previous study and proved appropriate to trigger different levels of emotions in each version [10].

After watching the videoclip, participants answered 6 questions to check if our manipulation worked (Additional file 1). The latter question consisted of a previously validated short version of the Positive and Negative Affect Schedule (I-PANAS-SF) scale [25, 26] that uses the negative items of the scale. Largely used in mood literature, the PANAS scale requires participants to report the extent to which they experienced a certain mood during a previous situation.

Subsequently, participants in the N and E conditions read a short description of the videoclip they had watched. Participants in the E-CR condition were asked to engage in cognitive reappraisal. Briefly, the instructions acknowledged that the participant might have found the video disturbing but benefits could also be extracted from the difficult situations one experiences and requested the participant to (i) look at the experience from a different perspective and try to identify how the resident in the video could have benefited from the it for his/her professional development, and (ii) write down at least one possible positive outcome from the experience.

#### Learning task

After completing the above-mentioned tasks (purportedly, Study 1) participants were requested to open the second envelope (purportedly, Study 2), which contained a 930-word text on oxidative damage during cardiac arrest. The text was prepared to by considering the participants’ prior knowledge to avoid ceiling effects and the relationship with the videoclip content (an incident with a patient undergoing cardiac arrest). The text was used in a previous study with similar participants [10], when it was prepared by two board-certified internists who have long-time experience in the residency program and familiarity with the residents’ knowledge level. The text proved sufficiently difficult in the previous study, when it was assessed by four 1^st^-year residents who were not participating in the study but were close to the participants in terms of their knowledge base.

Written instructions asked participants to read the text as they would usually read any chapter in a medical textbook, writing down the starting and ending time using a digital clock visible in the room. They were informed that they could use as much time as needed and, when finished, answered whether they already had knowledge of the content (Yes/No).

Subsequently, the participants filled in the Situational Cognitive Engagement Scale, an instrument that assesses involvement with learning tasks devised and validated by Rotgans and Schmidt [27] (Hancock’s coefficient H = 0.93 for exploration sample; H = 0.78 for cross-validation sample) (Additional file 2).

#### Testing task

After completing the learning task, the participants were requested to write down everything that they could remember from the text. Learning about a certain topic progresses through the enrichment of the semantic network of concepts related to that topic stored in the person’s memory. The more effective the learning, the more concepts could be recalled hence the number of concepts recalled offers a measure of learning [27–29].

Finally, participants provided demographic information.

### Data analysis

Sample uniformity between the three experimental conditions with respect to gender and age was checked by performing, respectively, a chi-square test and a one-way ANOVA. Participants’ familiarity with the situation portrayed in the videoclips (item #1 Additional file 1) and their judgment of the realism of this situation (item #2) were analysed by performing, respectively, a chi-square test and a one-way ANOVA.

Two measurements checked our manipulation: the average score of the two items of the questionnaire about the participant’s perception of the resident’s feeling (Additional file 1, items # 4 and %), and participant’s mean response to the negative items of the I-PANAS-SF scale (Chronbach’s Alpha, 0.74). For both measurements, the three experimental conditions were compared by performing a one-way ANOVA.

Two measurements of *learning processes* were analysed: time spent reading the medical text and the mean score obtained in the SCES (Additional file 2)(Chronbach’s Alpha, 0.67). Means for each experimental condition were computed and compared by performing separate one-way ANOVAs.

Finally, *learning outcomes,* as measured by the scores obtained in the recall test, were analysed. To obtain the score, an ‘answer key’ with all relevant concepts (84) present in the learning material prepared in a previous study was used [10]. One point was given to each concept mentioned, and the total number of points was the participant’s final score. First, two researchers (T.K.; M.P.T.N) independently and blindly coded 10% of the responses with a very high interrater agreement (ICC = 0.91), and the scoring proceeded with a single coder. Mean scores for each experimental condition were computed and compared by performing one-way ANOVA.

For all comparisons, the level of significance was set at *p* < 0.05. Statistical analyses were performed with SPSS version 25 for Mac.

## Results

Participants’ characteristics are presented in Table 1. No significant differences between the three conditions were found in gender, age, familiarity with the situation portrayed in the videoclip, or perceived realism of this situation.

**Table 1:** Participants’ gender and age, familiarity and realism of situation and manipulation check as a function of experimental condition (χ^2^(1, N = 220) = 0.44,*p* = 0.21)

	Neutral (N) (*N* = 31)	Emotional-without-antidote (E) (*N* = 34)	Emotional-with-antidote (E-CR) (*N* = 34)	Statistics
Sex (N, %)				χ^2^_(2, N = 99_) = 0.348, *p* = 0.840
Male	21 (68)	23 (68)	21 (62)	
Female	10 (32)	11 (32)	13 (38)	
Age (years), Mean (SD)^a^	25.52 (3.46)	25.32 (1.85)	25.44 (1.86)	F_(2, 96)_ = 0.050, *p* = 0.951
Familiar with the situation described in the video, N (%)	15 (48)	20 (59)	22 (65)	χ^2^_(2, N = 99)_ = 1.80, *p* = 0.406
Realism of video^b^, Mean (SD)	3.77 (0.76)	3.88 (0.73)	3.79 (0.88)	F_(2, 96)_ = 0.174, *p* = 0.840
Perception of resident’s emotions^b^, Mean (SD)	4.41 (0.37)	4.81 (0.27)	4.88 (0.22)	F_(2, 96)_ = 25.80, *p* < 0.001 N x E *p* < 0.001 N x E-CR *p* < 0.001 E-CR x E *p* = 0.63
PANAS scale^b^ Mean (SD)	2.77 (0.63)	3.40 (0.78)	3.56 (0.82)	F_(2, 96)_ = 9.87, *p* < 0.001 N x E *p* = 0.003 N x E_CR *p* < 0.001 E-CR x E *p* = 0.75

^*^ age ranged from 21 to 42 years

^a^ SD = standard deviation

^b^ range = 1 – 5

Two measurements showed that our manipulation worked. First, participants’ perceptions of the emotions portrayed in the video significantly differed between conditions. Post hoc comparisons with Gabriel correction showed the ratings to be significantly lower among participants who watched the neutral video (Table 1). Second, a significant difference between conditions was found in the scores obtained in the PANAS scale, with the score for the neutral condition significantly lower than those for the emotional conditions, which did not differ from one another (Table 1).

Table 2 presents the measurements of the learning process. Time spent studying the text was significantly lower (and similar) for participants from the two emotional conditions relative to the neutral condition. Ratings of the three conditions for the SCES did not significantly differ (Table 2).

**Table 2:** Learning process (time spent in studying the text and SCE) and learning outcomes (test score) measurements as a function of experimental condition

	Neutral (N) (*N* = 31)		Emotional-without-antidote (E) (*N* = 34)		Emotional-with-antidote (E-CR) (*N* = 34)		
	Mean	SD	Mean	SD	Mean	SD	Statistics
Time (min)	9.03	3.35	6.85	1.97	7.03	2.60	*F*_(2,96)_ = 6.53, *p* = 0.002, *η*_*p*_^2^ = 0.12 N x E *p* = 0.004 N x E-CR *p* = 0.01 E-CR x E *p* = 0.99
SCE (1 to 5)	3.34	0.59	3.13	0.77	3.09	0.76	*F*_(2,96)_ = 1.12, *p* = 0.332, *η*_*p*_^2^ = 0.23 N x E *p* = 0.57 N x E-CR *p* = 0.41 E-CR x E *p* = 0.99
Test Score	12.94	6.44	10.38	4.64	10.00	4.35	*F*_(2,96)_ = 3.030, *p* = 0.053, *η*_*p*_^2^ = 0.059 N x E *p* = 0.14 N x E-CR *p* = 0.07 E-CR x E *p* = 0.99

*n* Number of students that evaluated this version, *SD* Standard deviation, *SCE* Situational Cognitive Engagement

Finally, the learning outcomes are also presented in Table 2. The difference between the three experimental conditions failed to reach significance level. Participants from the E and E-CR conditions performed similarly, with lower (but not significantly) scores than those in the N condition.

## Discussion

This experimental study had a two-fold purpose: (1) examining whether the finding of a previous study [10] showing that negative emotions adversely affect medical residents’ learning of scientific information would replicate; (2) determining the effectiveness of an intervention designed to minimize the adverse effect, if any. Participants watched either a neutral or an emotional videoclip, the latter either followed by cognitive reappraisal or not. Subsequently, all participants studied a relevant medical text and took a recall test. Participants who watched the emotion-triggering video reported higher scores of emotions and spent less time studying the text than those in the neutral condition. The two emotional conditions performed similarly in the test, both lower than the neutral condition, but this difference was just above significance level.

What concerns the first study purpose, these results are in line with findings of a previous study that showed participants under the influence of negative emotions to invest less time to study the learning material and to learn less from it relative to peers in a neutral emotional state [10]. The difference in test scores between the emotional and the control conditions was just above the significance level, and no inferences can therefore be made regarding the learning outcomes, but a tendency can be noticed that replicates the pattern observed in other studies [6, 10]. In the present study, as in a previous one [10], self-reported cognitive engagement did not differ between conditions, which may reflect the limitations of self-report. The amount of time spent studying the text was substantially larger in the neutral condition than in the two emotional conditions, with a medium-large effect size [30]. The difference in study time per se may explain why the two emotional conditions tended to learn less since study time is known to directly influence learning [31]. However, the negative emotions may have also directly affected cognitive processes involved in learning [8]. Noteworthy, examining the mechanisms through which negative emotions affect learning was *not* among the present study aims, and though our findings suggest that an effect does exist, future research should investigate which specific elements of the learning process are affected by negative emotions.

Negative emotions have been shown to hinder undergraduate students’ learning [9]. Our findings suggest that, despite their increased experience, residents are still subject to the impact of emotions. Even though they were not personally involved in the situation displayed in the videoclip, the participants dedicated less time to study and tended to recall less from the scientific material. Real-life experiences would probably trigger stronger emotional reactions than those produced by vicarious experiences such as the one used in the study. If residents frequently encounter emotionally-charged situations, which is likely, their emotional reactions may hinder what they could gain from learning opportunities throughout the training. Considering the value of the residency training for professional development, this brings implication to both teachers and researchers. Teachers should be aware of potentially emotion-eliciting situations, be attentive to detect them and act supportively to make it easier for their residents to addressing these situations. To enable teachers to do this, researchers should direct their efforts to increase our understanding of the relation between emotions and learning and of when and how teachers could act upon emotional situations and their trainees’ reactions to them in order to optimize learning.

With regards to the second purpose of the study, the intervention designed to minimize the impact of negative emotions did not work out as expected. As a possible ‘antidote’ to the effect of negative emotions, we used cognitive reappraisal, which previous studies have shown to be an efficient emotional regulation tool [23, 32, 33]. Since both emotional conditions (with- and without-antidote) studied for a shorter time and performed similarly in the test, both tending to learn less relative to the neutral condition, the antidote failed to counteract the negative effect of emotions.

At least three possible explanations for this failure can be raised. A first explanation derives from the intensity of the emotional reaction to the video and the consequent engagement in the cognitive reappraisal effort [19]. Research shows that the use of ER strategies is associated with participants’ perception of stimuli intensity; generally, people compare their affective state to the desired one, engaging in ER when the difference is big enough [19]. In our study, a vicarious experience was used, and although ratings of emotions triggered by the emotional and neutral version of the video clip significantly differed, the low ratings in the PANAS scale suggest that emotions were not perceived as intense. Some participants may have not felt the need to fully engage in affect repair. Indeed, whereas some participants really described how the situation saw in the video could be reframed (e.g. “Facing adverse situations like the one showed in the video leads to learning of our limitations and the importance of reaching out to assistants…”) others simply mentioned a potential positive effect of that experience to the future (e.g. “resilience”). Such explanation linked to the insufficient intensity of the emotional reactions should be seen with cautious, however, in light of recent research on the “cognitive costs” of cognitive reappraisal [34]. It has been argued that employing reappraisal involves taxing cognitive processes more heavily than what was previously thought, especially as the emotional intensity situation increases [34, 35]. This recent research suggests that using cognitive reappraisal becomes more difficult in situations of high levels of emotional intensity [36], but such levels were not observed in our study.

A second possible explanation is that undetected ER strategies may have been employed by some participants in the emotional-without-antidote condition even without receiving instructions. Previous research describes adaptive ER as the flexibility to switch between different strategies and select the most appropriate one for the situation at hand [32]. An experimental study by Augustine and Hemenover [19] found individuals in the control group to show more efficient ER than those instructed to use a specific strategy. Participants may – consciously or not – use an ER strategy that has worked well for them before, whereas those instructed to use a specific strategy – in our study cognitive reappraisal – are forced to apply one that may not be successful for them.

A third possible explanation could be that the specific form of cognitive reappraisal used was inadequate. It could be that reframing the harsh treatment received by the resident in the videoclip, for example, as resulting from the committee members’ own vulnerabilities and poor self-control would result in better ER outcomes.

Further research should examine whether the cognitive reappraisal strategy would be effective when it is more strongly prompted, for example, because the situation provokes more intense emotional reactions. Another possible development would be to include a preliminary phase testing different cognitive reappraisal formulations. Different designs using the same strategy (cognitive reappraisal) before participants watch the video could give more insight on factors influencing its effectiveness. Furthermore, new studies with a similar experimental paradigm, should test different ER strategies.

This study has limitations. First, we used self-report to measure emotions, which raises questions about the participants’ awareness of their emotional states and their willingness to disclose them. Physiological measurements would have been more accurate, but they would reveal the measurement of emotional responses, potentially interfering with their free course. Second, we tested a convenience sample of residents from one school, which may have precluded adequate power to make borderline differences in the test scores reach the significance level and restricts generalization of the findings. Third, we assessed overall emotional status as a manipulation check without differentiating between discrete negative emotions, which may have affected sensitivity to detect differences between conditions, including regarding the effect of cognitive reappraisal. However, it can be questioned whether this would affect the results, considering the very close test scores of the two emotional conditions. Finally, we only examined knowledge acquisition as measured by a recall test, and it is unclear whether the findings apply to other types of learning, where cognitive reappraisal could help.

## Conclusion

Summing up, the present study replicates previous findings that negative emotions adversely impact medical residents’ subsequent learning. To get a better understanding of how to manage this effect, further research is called for. Apparently, these emotional states are harder to regulate than we assumed. Our ER intervention, although successfully applied in other domains, failed to diminish the negative effect of emotions on medical trainees’ learning. It is reasonable to hypothesise that real situations present an even bigger challenge, eliciting stronger negative emotions. This emphasizes the relevance of pursuing this line of investigation to reveal ER interventions suitable for medical education. Having access to an effective intervention would enhance trainees’ learning opportunities, their well-being and ultimately their ability to offer appropriate care to their patients.

## Supplementary Information

**Additional file 1.** Instructions (emotional-with-antidote condition). This is the questionnaire participants in the emotional-with-antidote condition were asked to fill in after watching the videoclip, which shows also the cognitive reappraisal formulation used in this study. 

**Additional file 2.** Situational Cognitive Engagement Scale. After the learning and testing phases, all participants answered these questions of the Situational Cognitive Engagement Scale, an instrument that assesses involvement with learning tasks. 

## Data Availability

The data analysed during the present study are available from the corresponding author upon reasonable request.

## Abbreviations

ER: Emotion regulation
N: Neutral (condition)
E: Emotional without antidote (condition)
E-CR: Emotional with antidote (condition)
SCES: Situational cognitive engagement scale
I-PANAS-SF: Positive and negative affect scale – short version

## Footnotes

1 Residents in Brazil have completed 6 years of the medical undergraduate program, obtaining a MD degree, and are physicians-in-training to obtain a specialty degree.
