# Supplementary material for: “Studying cognitive reappraisal as an antidote to the effect of negative emotions on medical residents’ learning: a randomized experiment”
Source: BMC Med Educ. 2023 Jan 28;23:72. doi: 10.1186/s12909-022-03996-2 (PMC9883942; doi:10.1186/s12909-022-03996-2)
Supplement: Supplementary file 1 — Additional file 1. Instructions (emotional-with-antidote condition). This is the questionnaire participants in the emotional-with-antidote condition were asked to fill in after watching the videoclip, which shows also the cognitive reappraisal formulation used in this study. [file 12909_2022_3996_MOESM1_ESM.docx]

**Appendix 1 – Instructions (emotional-with-antidote condition)**

Considering the video clip you watched, answer the following questions. In some questions you will see a scale. Please mark the score that best represent your impressions about the situation experienced by the resident in the video.

1. You (or someone you know) have already experienced a similar situation during your medical training? Remember, it does not have to be exactly the same situation, but an experience in which the resident sees himself or herself in a situation similar to the one portrayed in the video.

Yes No I am not sure

1. The video shows a realistic situation that is common during medical studies.

| 1 | 2 | 3 | 4 | 5 |
| --- | --- | --- | --- | --- |
| Totally disagree | Disagree | Do not agree nor disagree | Agree | Totally agree |

1. Do you think that watching this video helped you to understand the experience of the resident in the situation portrayed?

| 1 | 2 | 3 | 4 | 5 |
| --- | --- | --- | --- | --- |
| Not at all | To a small degree | Do not agree nor disagree | Yes, partially | Yes, very much |

1. Try to put yourself on the shoes of the first-year resident presented in this video and think about how he felt while listening to the comments of the assistant. The experience might have triggered strong emotional reactions in the resident.

| 1 | 2 | 3 | 4 | 5 |
| --- | --- | --- | --- | --- |
| Totally disagree | Disagree | Do not agree nor disagree | Agree | Totally agree |

1. Think about the residents’ feelings during the experience reported in this video. The resident might have gone through this experience with feelings that can be described as:

| 1 | 2 | 3 | 4 | 5 |
| --- | --- | --- | --- | --- |
| Deeply unpleasant | Unpleasant | Neutral | Pleasant | Very pleasant |

1. The words listed below indicate different feelings and reactions. Read each word and chose in the scale the number that indicates to what extension the situation showed in the videoclip you watched triggered that emotion in you

Upset

| 1 | 2 | 3 | 4 | 5 |
| --- | --- | --- | --- | --- |
| absolutely not | a little | moderately | a lot | extremely |

Hostile

| 1 | 2 | 3 | 4 | 5 |
| --- | --- | --- | --- | --- |
| absolutely not | a little | moderately | a lot | extremely |

Alert

| 1 | 2 | 3 | 4 | 5 |
| --- | --- | --- | --- | --- |
| absolutely not | a little | moderately | a lot | extremely |

Ashamed

| 1 | 2 | 3 | 4 | 5 |
| --- | --- | --- | --- | --- |
| absolutely not | a little | moderately | a lot | extremely |

Inspired

| 1 | 2 | 3 | 4 | 5 |
| --- | --- | --- | --- | --- |
| absolutely not | a little | moderately | a lot | extremely |

Anxious

| 1 | 2 | 3 | 4 | 5 |
| --- | --- | --- | --- | --- |
| absolutely not | a little | moderately | a lot | extremely |

Determined

| 1 | 2 | 3 | 4 | 5 |
| --- | --- | --- | --- | --- |
| absolutely not | a little | moderately | a lot | extremely |

Attentive

| 1 | 2 | 3 | 4 | 5 |
| --- | --- | --- | --- | --- |
| absolutely not | a little | moderately | a lot | extremely |

Scared

| 1 | 2 | 3 | 4 | 5 |
| --- | --- | --- | --- | --- |
| absolutely not | a little | moderately | a lot | extremely |

Active

| 1 | 2 | 3 | 4 | 5 |
| --- | --- | --- | --- | --- |
| absolutely not | a little | moderately | a lot | extremely |

“THERE ARE EVILS THAT COME TO GOOD”

You may have found the video disturbing because the resident was treated harshly. But there is always something good that can be drawn from difficult experiences. You know, "there are evils that come to good." Please look at the experience shown in the video from another perspective and try to identify how the resident may have benefited in terms of his professional growth. Name at least one possible positive outcome.

Please answer to the following questions about yourself

Age Gender

Year of end of graduation

Years working before start of residence
