# Supplementary material for: “Studying cognitive reappraisal as an antidote to the effect of negative emotions on medical residents’ learning: a randomized experiment”
Source: BMC Med Educ. 2023 Jan 28;23:72. doi: 10.1186/s12909-022-03996-2 (PMC9883942; doi:10.1186/s12909-022-03996-2)
Supplement: Supplementary file 2 — Additional file 2. Situational Cognitive Engagement Scale. After the learning and testing phases, all participants answered these questions of the Situational Cognitive Engagement Scale, an instrument that assesses involvement with learning tasks. [file 12909_2022_3996_MOESM2_ESM.docx]

**Appendix 2**: Situational Cognitive Engagement Scale

Before we proceed to the next phase, we would like to ask you to answer the next questions choosing the number that best express your situation when reading the text in the previous task:

1. “I was engaged with the task on hand”

| 1 | 2 | 3 | 4 | 5 |
| --- | --- | --- | --- | --- |
| Not true at all  for me | Not true  for me | neutral | True  for me | Very true  for me |

1. “I put in a lot of effort”

| 1 | 2 | 3 | 4 | 5 |
| --- | --- | --- | --- | --- |
| Not true at all  for me | Not true  for me | neutral | True  for me | Very true  for me |

1. “I wish I could still continue with the work for a while”

| 1 | 2 | 3 | 4 | 5 |
| --- | --- | --- | --- | --- |
| Not true at all  for me | Not true  for me | neutral | True  for me | Very true  for me |

1. “I was so involved that I forgot everything around me”

| 1 | 2 | 3 | 4 | 5 |
| --- | --- | --- | --- | --- |
| Not true at all  for me | Not true  for me | neutral | True  for me | Very true  for me |
